# Teaching space-group diagrams to chemistry students through a peer-tutoring approach

**DOI:** 10.1107/S2056989021008744

**Published:** 2021-08-27

**Authors:** Shao-Liang Zheng, Michael G. Campbell

**Affiliations:** a Department of Chemistry and Chemical Biology, Harvard University, 12 Oxford Street, Cambridge, MA, 02138, USA; b Department of Chemistry, Barnard College, 3009 Broadway, New York, 10027, USA

**Keywords:** upper-division undergraduate, graduate education, research, peer tutoring, collab­orative/cooperative learning, space group, symmetry, crystal structure

## Abstract

Peer tutoring is a teaching strategy that offers a creative way of getting students more involved and accountable for their own learning in college-level chemistry courses. The authors have found that the ‘*Symmetry and Space Group Tutorial*’ [Jasinski & Foxman (2007). *Symmetry and Space Group Tutorial*, V1.55. http://people.brandeis.edu/~foxman1/teaching/indexpr.html] lends itself well to a peer-tutoring approach in a crystallography course for chemistry students. This in-class activity provides an opportunity for students to learn space-group diagrams, understand basic symmetry concepts, organize what they have learned, and explain it to their peers, which leads to a deeper overall understanding of the subject. We report on our experience in planning peer tutoring, advise on best practices, and demonstrate the positive impact on student learning and engagement.

## Abstract 2

‘*Symmetry and Space Group Tutorial*’ (by Jerry P. Jasinski and Bruce M. Foxman) provides chemistry students an opportunity to learn space-group diagrams through the peer-tutoring approach.

## Introduction   

Peer tutoring is one of the most effective teaching strategies to engage students (Topping, 1996 ▸; Falchikov, 2001 ▸; Leung, 2015 ▸; McKeachie & Svinicki, 2015 ▸). ‘*To teach is to learn twice*’ – this approach provides a unique teaching opportunity that encourages students to get fully involved in their learning and to take responsibility for reviewing the course materials, understanding the basic concepts, and organizing their knowledge (Falchikov, 2001 ▸). As chemical crystallographers, one of our main educational missions is to make sure that chemistry students can engage with crystallography concepts and develop the skill sets they need to use crystallography effectively in their future research (Falvello, 2020 ▸). In the past decade, when teaching small-mol­ecule crystallography to graduate and upper-level undergraduate chemistry students, we have incorporated various active-learning methods, such as case-based learning and student-centered guest lecturing, in order to provide different learning paths to our students (Campbell *et al.*, 2016 ▸; Malbrecht *et al.*, 2016 ▸; Zheng *et al.*, 2018 ▸), and ensure they have a better understanding of found­ational crystallography concepts (Zheng & Campbell, 2018 ▸). When introducing space groups and their symmetry, a basic but complex topic, we have found that the well-written ‘*Symmetry and Space Group Tutorial’* (Jasinski & Foxman, 2007 ▸) can provide chemistry students with an opportunity to learn space-group diagrams through the peer-tutoring approach. Herein, guidelines are described for the peer-tutoring approach, developed for chemistry students from our teaching experiences, which may be of use to other educators in the field.

## Guidelines for peer tutoring approach   

The peer-tutoring approach is designed as an active learning experience in which the tutors, usually groups of two students, read through ‘*Symmetry and Space Group Tutorial’*, analyze an assigned space-group diagram copied from *Inter­national Tables for Crystallography* (Aroyo, 2016 ▸, 2021 ▸), organize and reformulate what they have learned, and then explain to their peers how to construct the assigned space-group diagram step by step using the traditional chalk-talk method. Because every member of the class has a chance to be the tutor as well as the student, such an arrangement maximizes the benefit to students and increases overall engagement.

### Timing   

We have found that it is best to arrange peer tutoring on space-group diagrams after students have been introduced to foundational crystallography concepts regarding crystal lattices, unit cells, Bravais lattices, and symmetry operations in crystals. We introduce the 17 plane groups to students first by using Escher drawings (Radaelli, 2011 ▸) because of the ease of more direct visualization (resources for demonstrating plane group symmetry using Escher drawings can be found in Table S1 in the supporting information). Students learn how to choose the correct unit cell as well as identify the asymmetric unit, space-group elements, space groups, and multiplicity (*Z*) in varied Escher drawings (such as *p*1, *p*2, *pg*, *p*2*gg*, *p*31*m*, and *p*3*m*1), and make connections to the plane-group diagrams in *Inter­national Tables for Crystallography* (Aroyo, 2016 ▸).

#### Format and schedule   

Students are instructed to read through the ‘*Symmetry and Space Group Tutorial’* the week prior to the in-class activity; it is important that students come to class prepared by having done the background reading. This excellent tutorial, combining both textual and visual reinforce­ment, explains things in a clear way so that most students with basic knowledge of point groups, unit cells, lattices, and space-group symmetry elements can understand. Thanks to the style of the tutorial – with elements of inquiry-based instruction, occasional humor, and links for the references bundled with the tutorial – we find that students come to class well-equipped to complete the activity and participate in discussions.

During the peer-tutoring activity, students are divided into groups of two, and each group is given a space-group diagram from *Inter­national Tables for Crystallography* (Aroyo, 2016 ▸) to examine together. The instructor explains the meaning of three-dimensional space-group symbols (*Lijk*) and provides several guiding questions to lead the students through the preparation of their chalk-talk (Fig. S1). Students are able to ask questions; however, it is best if the instructor acts only as a facilitator and re-directs students to the tutorial when possible.

After preparation, each group then holds a ten minute chalk-talk discussion to explain how to construct their assigned space-group diagram step by step to the rest of the class. They are also asked to identify the asymmetric unit, multiplicity (*Z*), and symmetry operations, and then answer any questions from the other students. The discussion that follows is usually focused on related concepts such as ‘*centrosymmetric space groups*’, ‘*non-centrosymmetric space groups’*, ‘*chiral mol­ecules enanti­opure’*, ‘*chiral mol­ecules racemate’*, ‘*chiral crystal structure*’, ‘*chiral (type) space group’* and ‘*space group of a chiral crystal structure’* (Flack, 2003 ▸; Thompson & Watkin, 2009 ▸). Examples of typical questions for the tutors include: ‘Why don’t we choose the *ab* projection for a monoclinic unit cell, just like the ortho­rhom­bic case?’; ‘Why is ‘*C*2_1_’ just the same space group as ‘*C*2′?’; ‘Why don’t mol­ecules in non-centrosymmetric space groups have to be enanti­omorphous?’.

### Post activities   

In order to evaluate and improve the effectiveness of the peer-tutoring activity, we ask students to fill out an anonymous feedback form after the chalk-talk (Fig. S2).

After this introduction to space groups and their symmetry in the lecture portion of the class, we usually also hold one lab session regarding how to use CSD software such as CSD *ConQuest* and *Mercury* (resources for using CSD *ConQuest* and *Mercury* can be found in Table S2). During the lab session, students also learn how to identify different space groups and visualize symmetry elements of a crystal from a *.cif* by using *Mercury* (Fig. S3). These hands-on activities further deepen students’ understanding and prepare them for the examination of structures from their own research projects in the future.

At the beginning of the following class, it is also important to review and highlight the key content from the students’ chalk-talks. This provides students with an opportunity to further reinforce their knowledge regarding the concepts that they developed during the peer-tutoring activity (Brown *et al.*, 2014 ▸).

## Further application   

This peer-tutoring approach for learning space groups has also been incorporated as a module in other undergraduate chemistry laboratory courses (Zheng & Campbell, 2018 ▸), as well as crystallography workshops and training sessions. During the COVID-19 pandemic, it was also adapted to a virtual setting for remote learning.

In our crystallography course, the peer-tutoring approach has also been applied in the context of learning disorder refinement. In this case, groups of tutors are assigned a particular disorder fragment, and then explain how to refine the disorder, including possible *SHELXL* commands added in the *.ins* file (Müller, 2006 ▸) and potential pitfalls to keep in mind during the refinement (Campbell *et al.*, 2016 ▸).

## Impact on student engagement and learning   

The peer-tutoring approach serves as an opportunity for students to learn that ‘*teaching is the best teacher*’. During the chalk-talk preparation, students can learn from the tutorial and practice the key concepts at their own pace. The open-ended guiding questions (Fig. S1) allow students to reformulate what they read and organize their talk in order to keep them engaged. This process aims to increase students’ feelings of responsibility and self-confidence. As one student pointed out in the anonymous feedback: ‘*never be afraid to look things up! If something is confusing to you, someone else in the past has also probably found it confusing, and there should be a solution out there*’. In this regard, the references embedded in the tutorial provide a great resource for students to work with. Rather than being a passive receiver of knowledge (Eilks & Byers, 2009 ▸), students are encouraged to actively showcase their ability to seek and collect information, and then efficiently share their knowledge with their peers. Indeed, the chalk-talk experience is not simply rote memorization and recall for students; the process of presenting and then answering questions requires the reconstruction of knowledge, which is a particularly effective way to increase content mastery (Karpicke & Blunt, 2011 ▸). Furthermore, peer tutoring can also accelerate learning for the other students who are not presenting, especially during the Q&A discussion. The discussions may sometimes go beyond the scope of the original chalk-talk because students feel encouraged to ask their peer tutors about weaknesses in their understanding. This is reflected in students’ anonymous feedback: ‘*what I was very confused by another student would have a wonderful and succinct way of explaining*’; ‘*one of Harvard’s strengths is its student body, and not to take advantage of that seems almost criminal’*.

## Conclusion   

Space-group symmetry is a basic but complex topic in small-mol­ecule crystallography education. A peer-tutoring activity using the ‘*Symmetry and Space Group Tutorial*’ offers a creative way to promote student learning and engagement, and achievement of the course learning goals. We suggest that such an approach may be broadly useful in crystallography education, and encourage other educators in the field to adopt and explore this method.

## Related literature   

The following references, not cited in the main body of the paper, have been cited in the supporting information: MacGillavry (1965 ▸); Schattschneider (2004 ▸). Online resources are also given in the supporting information.

## Supplementary Material

Supplementary figures and tables. DOI: 10.1107/S2056989021008744/dj2035sup1.docx
