# Supplementary material for: Teaching space-group diagrams to chemistry students through a peer-tutoring approach
Source: Acta Crystallogr E Crystallogr Commun. 2021 Aug 27;77(Pt 9):864–6. doi: 10.1107/S2056989021008744 (PMC8423007; doi:10.1107/S2056989021008744)
Supplement: Supplementary file 1 [file e-77-00864-sup1.docx]

Supporting information

1. Resources for demonstrating plane group symmetry using Escher drawings.

| Books | 1. MacGillavry, C.H. (1965). *Symmetry Aspects of M.C. Escher's Periodic Drawings*, IUCr, Utrecht.  2. Schattschneider, D. (2004). *Visions of Symmetry*. Harry Abrams, New York.  3. Radaelli, P. G. (2011). *Symmetry in Crystallography: Understanding the International Tables*. 1st ed.; Oxford University Press: London. |
| --- | --- |
| Online resources | 1. Escher Web Sketch. https://www.epfl.ch/schools/sb/research/iphys/teaching/crystallography/escher-web-sketch/ (accessed July 30, 2021).  2. Buseck, P. *From 2D to 3D: Escher Drawings — Crystallography, Crystal Chemistry, and Crystal “Defects”*  https://serc.carleton.edu/NAGTWorkshops/mineralogy/activities/25651.html (accessed July 30, 2021).  3. Escher, M. C. https://mcescher.com (accessed July 30, 2021). |

1. Resources for using CSD ConQuest and Mercury.

| Online resources | 1. CCDCCambridge. (2012). *How to: Show symmetry elements of a molecule using Mercury*. <https://www.youtube.com/watch?v=q219w2RC37o&list=PLEtBZ08SGISfk0GDaMnHy_VZW4g5leIXZ&index=3> (accessed July 30, 2021).  2. CCDCCambridge. (2020). *Introduction to ConQuest (CQ-001).* <https://www.ccdc.cam.ac.uk/support-and-resources/ccdcresources/Introduction-to-ConQuest-CQ001.pdf> (accessed July 30, 2021).  3. CCDCCambridge. (2020). *Visualisations (MER-001)*. <https://www.ccdc.cam.ac.uk/support-and-resources/ccdcresources/Structure_visualizations.pdf> (accessed July 30, 2021). |
| --- | --- |

**General Guiding Questions for space group chalk-talk**

1. How do you construct your assigned space group diagram? Why?
2. How do you choose the projection plane for your assigned space group?
3. How do you choose the origin for your assigned space group diagram?
4. How can you show that a *C*-centering operation involves a (½ ½, 0) translation applied to all primitive lattice points?
5. What is the “half translation rule” and how do you apply it?
6. What are the non-standard setting of the space groups? Why do we still use them?
7. What are the multiplicity (*Z*) and symmetry operations in your assigned space group? What is the possible chirality of a molecule crystalized in such a space group type?
8. General guiding questions for space group chalk-talk.

**Feedback for *space group* *chalk-talk***

1. Please name the primary things you have learned from the *space group chalk-talk* experience (max. of 3).
2. Please rate how much you learned about how *space groups and their symmetry* may help your research (1 = not much; 5 = very much), and explain.

| 1 | 2 | 3 | 4 | 5 | not applicable |
| --- | --- | --- | --- | --- | --- |
|  |  |  |  |  |  |

1. Would you recommend we continue similar *peer chalk-talk* in the future? How could it be improved?
2. An example of a feedback form for *space group chalk-talk*.

**Practice problems**

1. In Mercury, search for the crystal structure with reference code ZINJAH. What is the space group? ________

2. What symmetry element(s) are present in this unit cell?

3. The image below (left) is from the International Tables for X-ray crystallography for the space group of ZINJAH. Can you see all of the symmetry elements present using Mercury? (HINT: You may need to show more molecules in the lattice to see all of the symmetry elements)


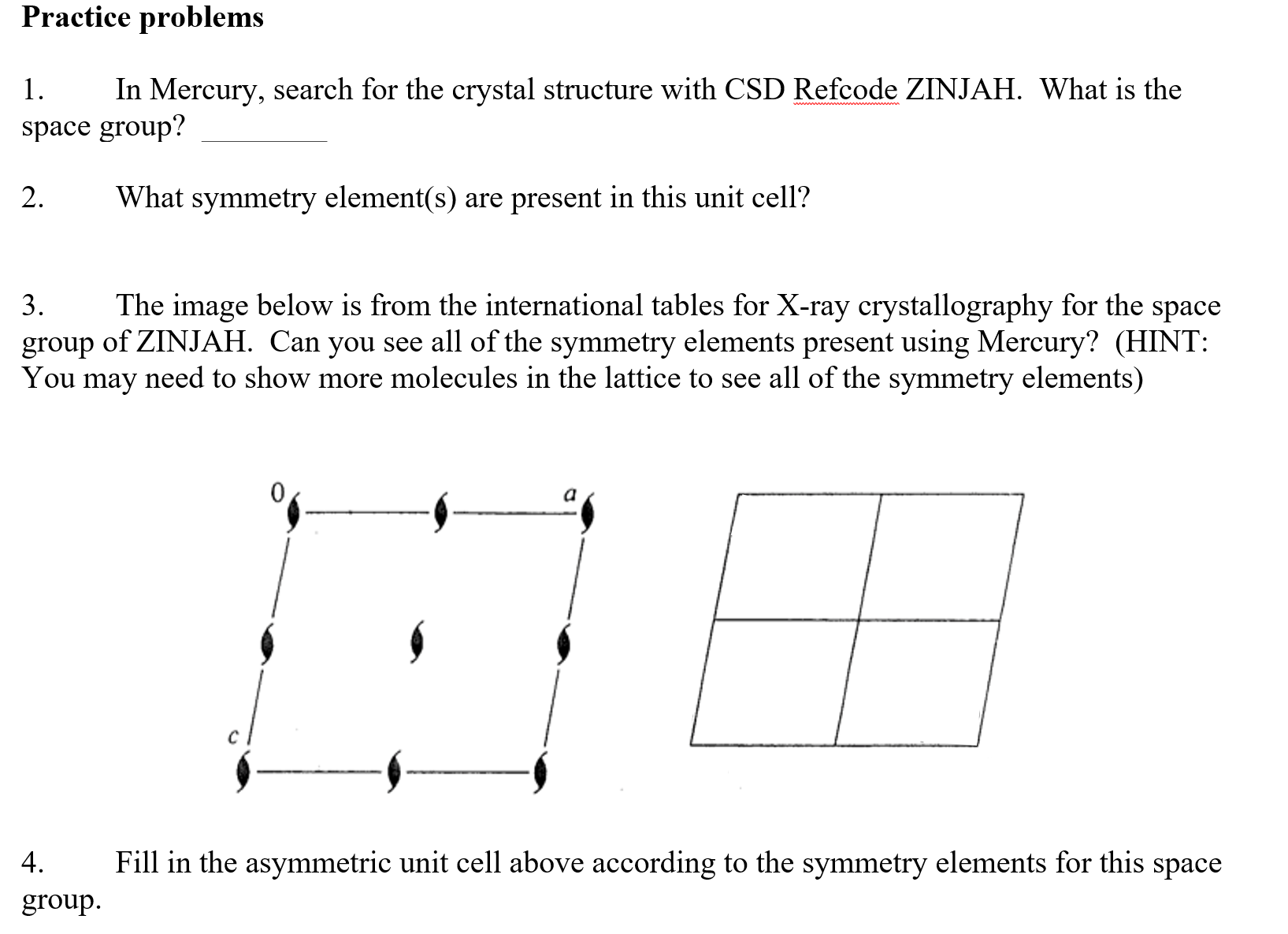


4. Fill in the asymmetric unit cell above (right) according to the symmetry elements for this space group.

Repeat questions 1-4 for the crystal structure with CSD Refcode FOYTAO. The symmetry elements for the space group of this structure are attached.


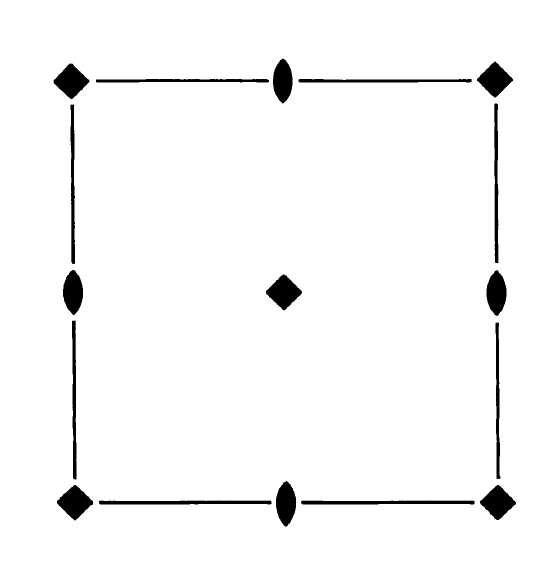

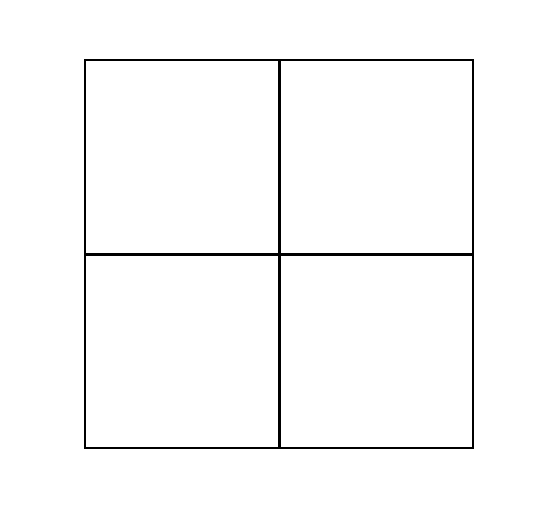


1. An example of *practice problems* using Mercury.
